# Experiences in a London Nursing Home and Also in the Pay Wing of a Metropolitan General Hospital

**Published:** 1913-05-31

**Authors:** 


### HOSPITALS FROM THE PATIENTS' POINT OF VIEW.

### Experiences in a London Nursing Home and also in the Pay Wing of a Metropolitan General Hospital.

It does not often fall to the lot of the mere lay patient to be privileged to describe his views and experiences whilst he was undergoing treatment, apart from the ordinary every-day treatment, which must, of necessity, be familiar to all. Nowadays so much is written and accomplished by the experts with the object of checking abuses and ameliorating the conditions appertaining to sickness, that it is hard to realise that the ultimate object of it all is to benefit the patient—in fact, the layman has cause to wonder whether the theorising would not be more effective if the patient were credited with perfectly legitimate views and opinions, and invited to express them more freely. One has only to spend a night in any large institution to realise that the "really good patient," in the hospital sense of the term, is he who is willing to subordinate his person and his predilections to the not-infallible wisdom of the authorities under whose care he seeks health and recovery.

My own experience is twofold. First, I underwent an operation for lithotrity in one of the best-known nursing homes in London, and some five years later I became a "nephrotomy case" as a paying patient in an institution connected with one of the largest general hospitals in the Metropolis. I do not claim any credit to myself for being a good patient. I am utterly intolerant of pain, and obsessed with the keenest desire to get back to my home comforts with the least possible delay; but some time has elapsed since my surgical escapades took place, and whatever reproachfulness or intolerance I may have felt at the time is obliterated by many months of comparative health and immunity from discomfort.

### Preliminaries before Operation.

In the nursing home I was immediately struck with the atmosphere of silent efficiency and good breeding which pervaded everything. Even the cordiality of my reception was tempered with a sense of discreet sympathy, which served to make me feel that after all there is some humanity left in those whose daily experiences might almost be expected to blunt their finer feelings. Such preparation as was necessary was carried out sensibly and without fuss until the time came for the performance of the operation. I was escorted by the nurse in whose charge I was placed to the lift, and eventually ushered into the "theatre," there to undergo the silly and superfluous formality of introduction to the surgeon's assistant and the anæsthetist. Seeing that I would never be likely in the ordinary course to see these gentlemen again, the mere fact that I was to surrender my consciousness to the ministrations of the one and my anatomy to the "assistance" of the other hardly warranted the necessity for an introduction. If I had succumbed I can imagine that they could have afforded to dispense with the handshake; as I recovered the handshake might, in deference to the surroundings, have been deferred to a more auspicious moment. Let that pass, however—worse was to come! I am tall and fairly agile, but to ask a man who is about to undergo his first operation to clamber upon the operation-table in the presence of the nurses, surgeons, and other occupants of the theatre, seemed to me to be the refinement of cruelty. Why could I not have been anæsthetised in a neighbouring room, instead of being expected, under most trying circumstances, to perform a not inconsiderable gymnastic feat? If only for that reason I would sooner run what risk there may be of sepsis and ill effects in surroundings costing less than eight guineas a week than submit to a similar ordeal a second time.

### Avoidable Miseries from the Traffic.

The operation was entirely successful; but how clearly I recall how much I suffered during the two nights that followed owing to the ceaseless din of the traffic in the stone-cobbled square beneath my window. I remember tossing about and wondering if it were possible that a single member of the governing body or surgical staff of that home had ever experienced the mental torture of incessant noise immediately after an operation. How foolish of them not to realise that the success of the Home would be immeasurably enhanced by coming to the best terms possible with the municipal authority with a view to replacing those granite setts with wood paving or some other less noisy substitute. I do not care to picture to myself the torture that a highly-strung patient must endure from that perpetual rattle if it has been his lot to undergo a severe operation and then be placed in a front room with the window open. After two or three days I was convalescent, and left the Home soon afterwards with feelings of gratitude to all who had come into contact with me, and a resolve, which has not yet borne fruit, to endeavour to remedy the anomalies referred to above.

### First Experiences at the Hospital.

Some five years later I was suddenly called upon to have a large stone extracted from my left kidney. There was no doubt about it; there it was, literally as large as life, in the *x*-ray photograph. For reasons into which it is needless to enter I decided to seek admission, as a paying patient, in an adjunct of a large general hospital. My own physician was to attend me, I had a slight acquaintance with the resident medical officer, and a surgeon was selected whose qualifications were unimpeachable. By dint of playing my cards properly I was absolved from the necessity of entering what I will call, for the sake of brevity, the Home until the afternoon before the operation took place; and I here wish to make it clear that the sister-in-charge was away on a holiday at the time, the charge-nurse undertaking her duties in the meantime. I arrived at the Home and was some minutes wandering about before finding any-one to report myself to. My bed was already allotted to me, and after unpacking my scanty wardrobe, as nobody seemed to want me, I set out on a tour of apprehensive exploration.

### Faulty Nursing Procedure.

Eventually a nurse sought me out, and in a guileless way offered me a drink, of which I partook with the direst results. I was left severely alone until supper, which I ate in solitary state. At 7.45 p.m. I was ordered to bed, fully conscious by this time that institutional procedure takes no count of cases until they become interesting, and that paying patients' privileges would appear to be hardly bought. Next morning the same mysterious aloofness was observed, save only for a hurried visit from the physician and the surgeon; the charge-nurse followed, and, after satisfying herself that no local preparation was necessary, returned, and, having muttered something about an injection, proceeded to administer, with what seemed an interminable needle, an extremely adequate hypodermic dose of colouring matter which literally made me sit up, so totally unprepared was I for anything of the kind.

### The Second Operation and After.

I was spared the introduction to the anæs-thetist and the "assistant," and, thanks to enlightened methods, avoided that interminable "going off" process and the encounter with the operating-table. I recovered consciousness in a state of tremendous heat, due, so I learned after-wards, to the fact that the nurse or nurses had been far too generous in their conception of my hot-water bottle needs. I luckily escaped, so far as I can recollect, any serious discomfort from the after-effects of the anæsthetic; but I am bound to record that it passes my comprehension that modern scientific knowledge, as revealed at the Home, should have been utterly incapable of alleviating the misery caused by flatulence, which I underwent for at least three days after the operation.

### Post-operative Discomfort.

Surely it would only be necessary for the Presidents of the Royal Colleges of Physicians and Surgeons to undergo one or two severe abdominal operations for this evil to be promptly and effectively avoided. I tried enemas of three different kinds and was given peppermint water to drink, as if to remind me of the futility of modern methods, but to no avail, and had to console myself with the information that "this always happens with abdominal cases, and will be all over in three or four days." Nor was this all. I could not sleep, and at length bromide was prescribed, but as this was administered in the form of a crystal-like con-coction almost devoid of fluid, my feelings can be better imagined than described.

### Absence of Trained Nurses; Tormenting Noises.

The Home advertised that the nurses were trained—that this was not the case, at any rate in a large number of cases, I can testify with confidence. For example, not having had my dressing renewed for about three or four hours, and being in a state of extreme dis-comfort in consequence, I rang my bell one evening *for over an hour* without getting any reply, and when I did succeed in attracting attention was told that the nurse under whose charge I was placed had been going in for an examination, and there was no one to take her place! The bed urinals were so small that I feel sure that they must have been designed originally for children or persons whose powers of micturition were suspected of serious defect. The noise within and without the Home was perpetual, and not always unavoidable. An officer of the institution, who inhabited a wing near by, took the precaution of obtaining double-windows in apparent oblivion of the fact that the paying patients were ill and needed this precaution infinitely more than he. Engineering skill cannot surely confess to an inability to supply a thorough supply of aseptic air without looking to the outside windows for assistance. As regards the internal noise, however ill one may feel, one cannot cavil at the rattle of the dressing appliances on the trolley as it goes its rounds, but it passes the bounds of comprehension that administration should be so utterly inefficient as to permit a patient to be wheeled over stone paving on a bed which is innocent of rubber-tired castors.

### "The Soulless Occupant of a Bed."

As convalescence brought the day of de-parture nearer one began to realise that if one goes to an institution, either as an ordinary patient or as an inmate of the pay ward, one must not expect to be treated during the period immediately preceding and following the operation as more than "a case"—the soulless occupant of a bed—to be run according to prescribed and exact rule and discipline—powerless to elicit any sense of the humanities—ineligible to expect it. Nor is this to be wondered at, seeing that the night duty for some twenty patients in varying stages of recovery was entrusted to two young nurses who, however willing, could not attend to more than one summons a-piece at any given moment.

### Two Good Suggestions.

One word more. I would suggest two important reforms in our institutional world. First, that every patient on or after leaving should be cordially invited to relate any grievances he may have to the governing body in the certainty that they will be examined, and, if possible, redressed; and, secondly, that the authorities of hospitals and nursing homes should be everlastingly impressed with the vital importance of letting no opportunity pass of satisfying themselves that externally and internally the utmost quiet is secured at whatever cost.

